# CRABS CLAW Acts as a Bifunctional Transcription Factor in Flower Development

**DOI:** 10.3389/fpls.2018.00835

**Published:** 2018-06-20

**Authors:** Thomas Gross, Suvi Broholm, Annette Becker

**Affiliations:** ^1^Department of Biology, Institute of Botany, Justus Liebig University Giessen, Giessen, Germany; ^2^Biosciences and Environment Research Unit, Academy of Finland, Helsinki, Finland

**Keywords:** CRABS CLAW, carpel, protein domains, *Arabidopsis thaliana*, YABBY

## Abstract

One of the crucial steps in the life cycle of angiosperms is the development of carpels. They are the most complex plant organs, harbor the seeds, and, after fertilization, develop into fruits and are thus an important ecological and economic trait. CRABS CLAW (CRC), a YABBY protein and putative transcription factor, is one of the major carpel developmental regulators in *A. thaliana* that includes a C2C2 zinc finger and a domain with similarities to an HMG box. CRC is involved in the regulation of processes such as carpel fusion and growth, floral meristem termination, and nectary formation. While its genetic interactions with other carpel development regulators are well described, its biochemical properties and molecular way of action remain unclear. We combined Bimolecular Fluorescence Complementation, Yeast Two-Hybrid, and Yeast One-Hybrid analyzes to shed light on the molecular biology of CRC. Our results showed that CRC dimerizes, also with other YABBY proteins, via the YABBY domain, and that its DNA binding is mainly cooperative and is mediated by the YABBY domain. Further, we identified that CRC is involved in floral meristem termination via transcriptional repression while it acts as a transcriptional activator in nectary development and carpel fusion and growth control. This work increases our understanding on how YABBY transcription factors interact with other proteins and how they regulate their targets.

## Introduction

Floral development and especially carpel development is one of the most crucial steps in the life cycle of flowering plants, since it is the prerequisite for reproduction and, subsequently, the formation of seeds and fruits. The orchestration of carpel development requires a large number of physically and genetically interacting transcription factors that are members of a several different transcription factor families (Reyes-Olalde et al., 2013). One central regulator of carpel development genetically linked with many others is CRABS CLAW, a YABBY transcription factor. Most members of this family act in regulatory networks together with members of the HD-ZIP III family to regulate adaxial-abaxial polarity in lateral plant organs, e.g., leaves, carpels, and ovules (Sablowski, 2015; Tatematsu et al., 2015).

The plant-specific YABBY proteins with only six members in *Arabidopsis thaliana* [CRC, FILAMENTOUS FLOWER (FIL), INNER NO OUTER (INO), YABBY2, 3, and 5] are transcription factors with a C2C2 zinc finger domain and the family specific YABBY domain, a modified HMG box with a basic helix-loop-helix secondary structure. Identified by Bowman and Smyth (1999), it was named after the crabs claw like appearance of the apically unfused carpels of the *crc-1* mutant. Han et al. (2012) showed that CRC is able to bind to DNA, in particular promoter regions of *3-KETOACYL-COA SYNTHASE 7* and *15* (*KCS7* and *KCS15*), two genes that are involved in the synthesis of very-long-chain fatty acids which are then used as signaling molecules or in cuticular wax synthesis (Joubès et al., 2008). However, CRC’s DNA binding motif or target genes involved in carpel developmental processes remain unknown.

The *crc-1* mutant shows shorter and wider gynoecia with the two carpels being unfused at the apex and flowers lacking nectaries (Bowman and Smyth, 1999). The lack of carpel fusion is intensified in double mutants of *SPATULA* (*SPT*) and *CRC* in which the two carpels fuse only at their base (Alvarez and Smyth, 1999, 2002). All YABBY genes are expressed in flowers but only *CRC* and *INO*’s expressions are restricted to flowers, to carpels and ovules, respectively. *CRC* and *INO* regulate, in combination with *KANADI* (*KAN*) genes and *ETTIN (ETT)*, adaxial-abaxial patterning of the carpels and of the developing ovules by specifying the abaxial side (reviewed in Sablowski, 2015). Furthermore, the action of adaxial regulators, like members of the HD-ZIP III family is suppressed, maybe by activating the transcription of miR165/166 (Tatematsu et al., 2015).

The *crc-1* mutant shows a weak meristem indeterminacy phenotype and thus a mild surplus in carpels (Alvarez and Smyth, 1999; Bowman and Smyth, 1999; Alvarez and Smyth, 2002). A more detailed analysis revealed that *CRC* acts redundantly with *REBELOTE* (*RBL*), *ULTRAPETALA 1* (*ULT1*), and *SQUINT* (*SQN*) in floral meristem termination (Prunet et al., 2008). The loss of floral meristem determinacy is at least partially caused by a reduced expression of *AGAMOUS* (*AG*) in double mutants of these three genes with *CRC* (Prunet et al., 2008). While, *RBL*, *ULT1*, and *SQN* have been shown to act epistatic to *AG*, *CRC* is a direct target of *AG* (Gomez-Mena et al., 2005; Lee et al., 2005; Ó’Maoiléidigh et al., 2013). AG and other MADS box transcription factors, like PISTILLATA (PI), APETALA 1 (AP1), and APETALA 3 (AP3) directly bind to the *CRC* promoter. They either activate, as in the case of AG, or repress as in the case of PI, AP1, and AP3 *CRC* expression in the developing flower and restrict *CRC* expression to nectaries and carpels (Gomez-Mena et al., 2005; Lee et al., 2005; Ó’Maoiléidigh et al., 2013).

This regulation of CRC transcription causes a highly complex spatial and temporal expression pattern inside the developing carpels (Bowman and Smyth, 1999; Alvarez and Smyth, 2002). *CRC* expression starts in stage 6 (staging according to Smyth et al., 1990) and continues to stage 7 –8 in two distinct domains of the carpels: In the epidermis of the carpels, surrounding the circumference, and in four interior stripes adjacent to the developing placenta (Bowman and Smyth, 1999; Alvarez and Smyth, 2002; Lee et al., 2005). The expression in the four stripes forms a basal-apical gradient and decreases in later floral stages, whereas, the epidermal expression ceases in the future replum but is maintained in the valves until the mid of stage 12 (Bowman and Smyth, 1999; Alvarez and Smyth, 2002; Lee et al., 2005).

CRC orthologs from other angiosperms show additional functions suggesting a complex molecular evolution of the protein (Fourquin et al., 2007; Orashakova et al., 2009). The *Oryza sativa CRC* ortholog *DROOPING LEAF* (*DL*) regulates carpel identity, floral meristem termination, and leaf midrib formation (Yamaguchi et al., 2004; Ishikawa et al., 2009). In the basal eudicot *Eschscholzia californica*, a knock-down of *EcCRC* leads to a loss of floral meristem determinacy, impaired replum formation, and a reduced seed set, due to malformed placenta tissue (Orashakova et al., 2009). Similar to this, a knock-down of *PsCRC* in *Pisum sativum* impaired carpel fusion and the seed set was reduced due to stigma and style malformations (Fourquin et al., 2014). However, while the CRC phenotype and its genetic interactions have received considerable attention over the past decades the molecular functions of CRC protein domains are unknown as well as the molecular mechanism of its action. Here, we assign functions to CRC’s protein domains and show that CRC acts through activating regulatory processes during carpel development.

## Materials and Methods

### Localization of the CRC Protein

**FIGURE 1:**
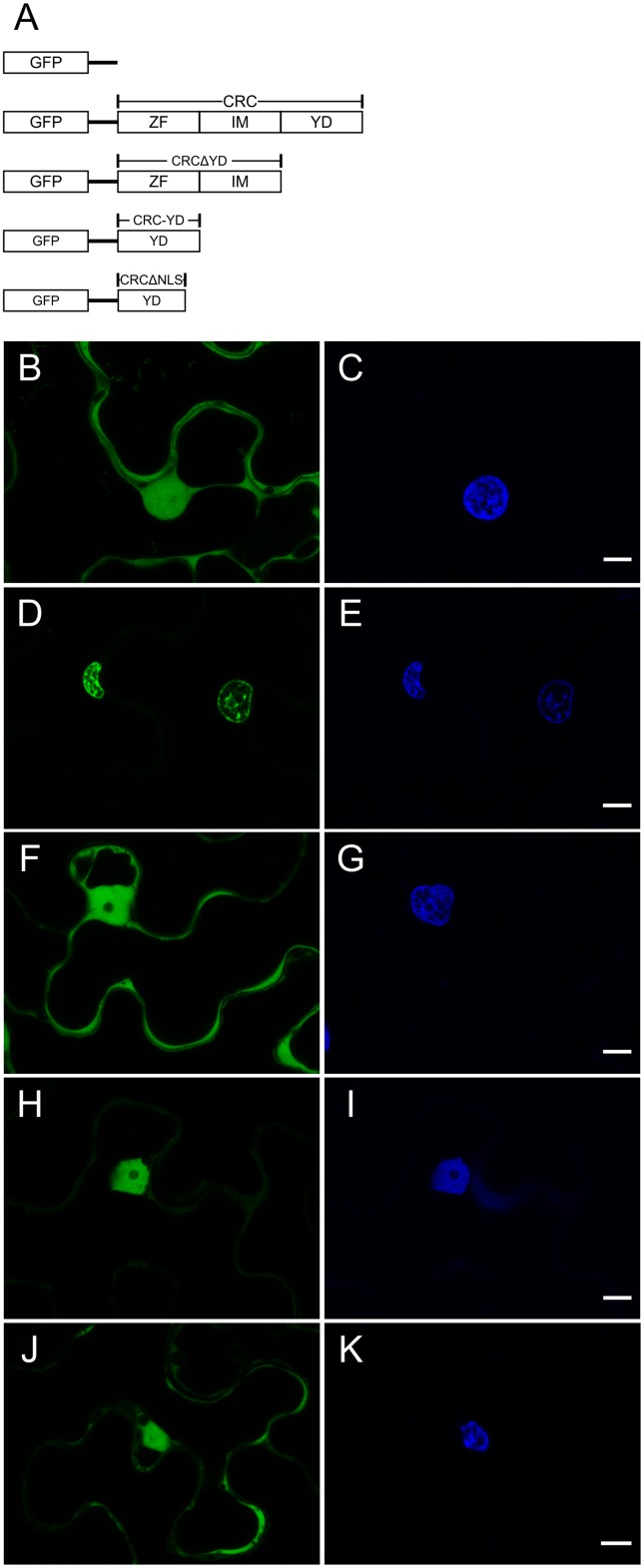
Analysis of the intracellular localization of GFP::CRC employing different CRC deletion variants. GFP fusion proteins were detected by CLSM. False colors were assigned to GFP (green, left panels) and DAPI (blue, right panels) which stains DNA. **(A)** Schematic representation of the GFP-CRC constructs used in this study. **(B,C)** 35S::GFP (29.26 kDa), **(D,E)** 35S::GFP::CRC (48.97 kDa). **(F,G)** 35S::GFP::CRCΔYD (40.75 kDa); **(H,I)** 35S::GFP::CRC-YD (34.75 kDa); **(J,K)** 35S::GFP::CRCΔNLS (47.97 kDa). ZF, zinc finger domain; IM, intermediate domain; YD, YABBY domain; NLS, nuclear localization signal. All scale bars represent 10 μm.

The full length coding sequence (CDS) of *CRC*, *CRC* deletion constructs *CRCΔZinc Finger*, (*CRCΔZF*) *CRCΔIntermediate* (*CRCΔIM*), *CRCΔYABBY* (*CRCΔYD*), and single domains *CRC Zinc Finger* (*CRC-ZF*), *CRC Intermediate* (*CRC-IM*), *CRC YABBY* (*CRC-YD*), *CRC-YDΔnuclear localization signal* (*CRC-YDΔNLS*), and *CRC-NLS* were cloned as EcoRI/BamHI fragments (for primer sequences see Supplementary Table 1, and for length of the individual fragments see **Figure 1A** and Supplementary Figure 2A) into the N-terminal GFP fusion vector pEGAD (Cutler et al., 2000). The borders of the CRC domains (ZF aa 26–53, IM aa 54–108, YD aa 109–155) are based on the classification of Bowman and Smyth (1999). As Bowman and Smyth (1999) predicted only the secondary structure of the YABBY domain, we performed a secondary structure prediction of full length CRC with REPPER (Jones, 1999; Gruber et al., 2005; Zimmermann et al., 2017) to identify additional secondary structures. To identify CRC’s nuclear localization signal, single amino acids in the core region of CRC’s putative nuclear localization signal were substituted via site directed mutagenesis (Hemsley et al., 1989). The *CRC* versions encoding for CRC K110T, P111A, P112A, E113G, K114T, K115T, Q116L, and R117T were introduced into the GreenGate system [the plasmid kit used for generation of plant transformation constructs was a gift from Jan Lohmann (Addgene kit # 1000000036)] and tagged with C-terminal *GFP* under the control of the CaMV 35S promoter (Lampropoulos et al., 2013). The *CRC* versions were transiently expressed in leaves of 4 weeks old *Nicotiana benthamiana* plants after infiltration with *Agrobacterium tumefaciens* GV3101, harboring the respective *CRC* version. For GreenGate based plasmids, *A. tumefaciens* GV3101 pSOUP^+^ was used. Small leaf disks were excised and stained with 1 ng/ml DAPI 2 days after infiltration. Microscopical analysis was performed with the Leica fluorescence microscope DCM5500 (Leica Microsystems GmbH, Wetzlar, Germany) using A4 filter for DAPI fluorescence and L5 filter for GFP fluorescence, or with Leica TCS SP8 confocal laser scanning microscope with excitation at 405 nm for DAPI and 488 nm for GFP, and a detection range of 413 – 460 nm for DAPI fluorescence and of 496 – 569 nm for GFP fluorescence.

### Yeast One-Hybrid Analysis (Y1H)

Parts of the promoter regions of *KCS7* and *KCS15* from *A. thaliana* Ler-0 (Han et al., 2012) were cloned into the Y1H vector pAbAi (Takara Clontech, Saint-Germain-en-Laye, France) using HindIII and SacI restriction sites. The assembled pAbAi *proKCS7* and pAbAi *proKCS15* constructs were linearized with BstBI and transformed into *Saccharomyces cerevisiae* Y1HGold according to the Yeast Transformation System 2 manual (Takara Clontech). Clones carrying the desired DNA fragment were screened for auto activation on synthetic uracil dropout medium, supplemented with 100 ng/ml, 150 ng/ml, 200 ng/ml, 500 ng/ml, and 1000 ng/ml Aureobasidin A (Takara Clontech). Bait strain colonies that showed no auto activation were selected and were transformed with prey plasmids: full-length *CRC*, deletion constructs, and single domains in pGADT7 (Takara Clontech), according to the modified Yeast Transformation System 2 manual. Additionally, three CRC mutant versions were generated via site directed mutagenesis, in which either the zinc finger or the YABBY domain was non-functional, and a combination of both: CRC zinc finger mutated (CRC ZFm), with C26W and C29W; CRC YDm with A121P, N123P, M126P, E129P, R132P, F146P, A149P, N152P, and K155P; and the combination: CRC ZFm/YDm.

### Yeast Two-Hybrid Analysis (Y2H)

Protein interaction tests via Y2H were carried out as described in Lange et al. (2013). The CDS of *YABBY 3* (*YAB3*), *INO*, and *CRC* were translationally fused to the GAL4 activation domain (AD) or GAL4 DNA binding domain (BD) by cloning them into pGADT7 and pGBKT7, respectively. The yeast 2-hybrid vectors of *FILAMENTOUS FLOWER* (*FIL*), *YABBY 2* (*YAB2*), and *YABBY 5* (*YAB5*) were a kind gift of John Golz (Stahle et al., 2009). Transformation of *S. cerevisiae* AH109 was carried out as described (Kaufmann et al., 2005). Growth assays on synthetic leucine, tryptophan, and histidine dropout medium (SD-Leu/-Trp/-His), supplemented with 3 mM 3-Amino-1,2,4-triazole (3-AT), and MEL1 based α-galactosidase assay was carried out in triplicates. As positive control, a combination of AD-*EcSEIRENA*/BD-*EcDEFICIENS2* (Lange et al., 2013) was used and a combination of the empty vectors pGADT7/pGBKT7 as negative control.

### Bimolecular Fluorescence Complementation (BiFC)

Bimolecular fluorescence complementation assays to test for homodimerization of CRC were performed as described in Lange et al. (2013). The different *CRC* variants (*CRC*, *CRCΔZF*, *CRCΔIM*, *CRCΔYD*, *CRC-ZF*, *CRC-IM*, and *CRC-YD*) were cloned into the respective pNBV vector as described above (Walter et al., 2004; Lange et al., 2013) and the expression cassettes were transferred to the plant transformation vector pMLBART and transformed into *A. tumefaciens* GV3101. Leaves of 4 weeks old *N. benthamiana* plants were inoculated with the *A. tumefaciens* strains, expressing the proteins to be tested, according to Lange et al. (2013). Small leaf disks were excised and stained with 1 μg/ml DAPI 2 days after infiltration. Microscopical analysis was performed with Leica fluorescence microscope DCM5500 using A4 filter for DAPI fluorescence and L5 filter for GFP fluorescence.

### *A. thaliana* Manipulation

The CDS encoding the EDLL domain (Tiwari et al., 2012) and the SRDX domain (Hiratsu et al., 2003) were synthesized as oligonucleotides and introduced into the GreenGate system. Using the GreenGate system, *proUBQ10:N-Dummy:CRC:C-Dummy:tUBQ10; proMAS:BASTA:tMAS*, *proUBQ10:N-Dummy: CRC:SRDX:tUBQ10; proMAS:BASTA:tMAS*, and *proUBQ10:N-Dummy:CRC:EDLL:tUBQ10; proMAS:BASTA:tMAS* were assembled according to Lampropoulos et al. (2013). Plasmids verified by sequencing were transformed into *A. tumefaciens* GV3101 pSOUP^+^. All constructs were introduced into *A. thaliana* Ler-0 plants via floral dip (Davis et al., 2009). Putatively transgenic seeds were grown on soil and selected by spraying with 300 μM Basta (Bayer CropScience Deutschland GmbH, Langenfeld, Germany) 7 days after germination with repetitions on every second day. Basta resistant seedlings were further genotyped by PCR. Expression strength was analyzed via qRT-PCR. Total RNA from leaves and of buds of representative wild type, *crc-1*, CRC over expression (CRCoe), CRC-SRDX, and CRC-EDLL was isolated in triplicates using the NucleoSpin RNA Plant kit (Macherey-Nagel GmbH & Co., KG, Düren, Germany) and transcribed into cDNA using RevertAid H Minus Reverse Transcriptase (Thermo Fisher Scientific Inc., Schwerte, Germany) with random hexamer primer. The cDNA was diluted 1:50 and added to Luna master mix (NEB Inc., Frankfurt am Main, Germany), followed by a PCR run using Lightcycler 480 II (Roche Diagnostics Deutschland GmbH, Mannheim, Germany) to detect *CRC* expression in leaves and *KCS7* and *KCS15* expression in buds. Afterward, the obtained data was analyzed according to Pfaffl (2001) and Taylor et al. (2010).

### YABBY Binding Motif Analysis

The promoter regions of *BLADE ON PETIOLE1* (*BOP1*, AT3G57130), *BOP2* (AT2G41370) *POLYUBIQUITIN10* (*UBQ10*, AT4G05320), *GLYCERALDEHYDE-3-PHOSPHATE DEHYDROGENASE C-2* (*GAPDH*, AT1G13440), and *ELONGATION FACTOR 1* α (*EF-1*α, AT1G07920) were screened for the presence of the four YABBY binding motifs (Shamimuzzaman and Vodkin, 2013; Franco-Zorrilla et al., 2014), using PlantPAN (Chow et al., 2016).

## Results

### CRC’s Nuclear Localization Requires the YABBY Domain

The CRC protein includes a single C2C2 type zinc finger and the YABBY domain which shows weak similarities to protein domains found in HMG (high mobility group) proteins (Bowman and Smyth, 1999). Secondary structure prediction (Supplementary Figure 1) by REPPER (Gruber et al., 2005; Zimmermann et al., 2017) suggests that the zinc finger (between amino acid position 26–53) is formed by two neighboring α-helices (red) flanked by two β-strands (green). The intermediate segment (between position 54–108) is predicted to contain two β-strands that are followed by an unstructured stretch of amino acids that may serve as flexible linker region and the YABBY domain (between position 109–155) is predicted to include one short and two long α-helices.

The presence of a zinc finger, which may bind DNA, RNA, or proteins (as reviewed in Burdach et al., 2012), suggests that CRC is a transcription factor, a hypothesis supported also by its DNA binding capacity (Han et al., 2012). We aimed to support this hypothesis by studying CRC’s intracellular localization (**Figure 1**). The full-length open reading frame (ORF) of CRC, several deletion constructs, and the individual domains were translationally fused to GFP and driven by the CaMV 35S (35S) promoter (**Figure 1A** and Supplementary Figure 2A). These constructs were transiently expressed in *N. benthamiana* epidermal cells to observe the intracellular localization of the encoded protein fusions by confocal-laser-scanning (**Figure 1**) and fluorescence microscopy (Supplementary Figure 2). As a control, native GFP driven by the 35S promoter was found to be present in both, the cytoplasm and nuclei (**Figure 1B**), as a comparison with the DAPI staining indicates (**Figure 1C**) but was absent in other organelles like the vacuole. In contrast, GFP::CRC was localized exclusively to the nuclei of the leaf epidermal cells (**Figures 1D,E**), supporting the hypothesis that CRC acts in the nucleus.

To identify the protein domain required for nuclear localization of CRC, we truncated the protein and used both, individual domains and deletion constructs for further localization studies (**Figure 1A** and Supplementary Figure 2A). The deletion of the YABBY domain led to an accumulation of GFP::CRCΔYD in the cytoplasm, similar to native GFP (**Figures 1F,G**). The removal of the zinc finger or of the serine-proline rich intermediate domain from the full-length protein instead resulted in the respective fusion protein being confined to the nucleus with no fluorescence detected in the cytoplasm (Supplementary Figures 2B–E). The localization of the individual zinc finger and of the intermediate domains showed the same subcellular localization as native GFP, throughout the cytoplasm and the nuclei (Supplementary Figures 2F–I).

Since the deletion of the YABBY domain increased the amount of GFP::CRCΔYD in the cytoplasm, we further analyzed the YABBY domain. When GFP was fused to only the YABBY domain, the fusion protein showed a restored nuclear localization (**Figures 1H,I**). This suggests that CRC’s NLS is located in the YABBY domain. With the position of the NLS restricted to the YABBY domain, we compared the predictions about the position of the NLS of Bowman and Smyth (1999) with known NLS consensus sequences (Kosugi et al., 2008) and identified eight possible amino acid residues in the N-terminal part of the YABBY domain that might be part of the NLS. When we removed these eight residues from the YABBY domain (GFP::CRCΔNLS), the exclusive nuclear transport stopped and the fusion protein accumulated in the cytoplasm like the native GFP (**Figures 1J,K**). However, if only these eight amino acid residues were fused to GFP (Supplementary Figures 2J,K), the distribution of the fusion protein was identical with native GFP, and thus, the eight residues alone, were insufficient to induce exclusive nuclear import. We were then interested in analyzing the NLS sequence in more detail and produced single amino acid substitutions of these eight amino acids. The eight CRC versions with single substitutions (CRC K110T, P111A, P112A, E113G, K114T, K115T, Q116L, and R117T) were fused to GFP and transiently expressed in *N. benthamiana* leaves. However, none of the single amino acid substitutions changed the localization of the respective CRC fusion variants and they all remained inside the nuclei (Supplementary Figure 3).

In summary we find that the nuclear localization of the CRC protein depends on the presence of the YABBY domain. However, a conventional NLS as predicted by Kosugi et al. (2008) which can be abolished by single amino acid exchanges does not reside in the YABBY domain.

### CRC Forms Homodimers and Heterodimers With INO

**FIGURE 2:**
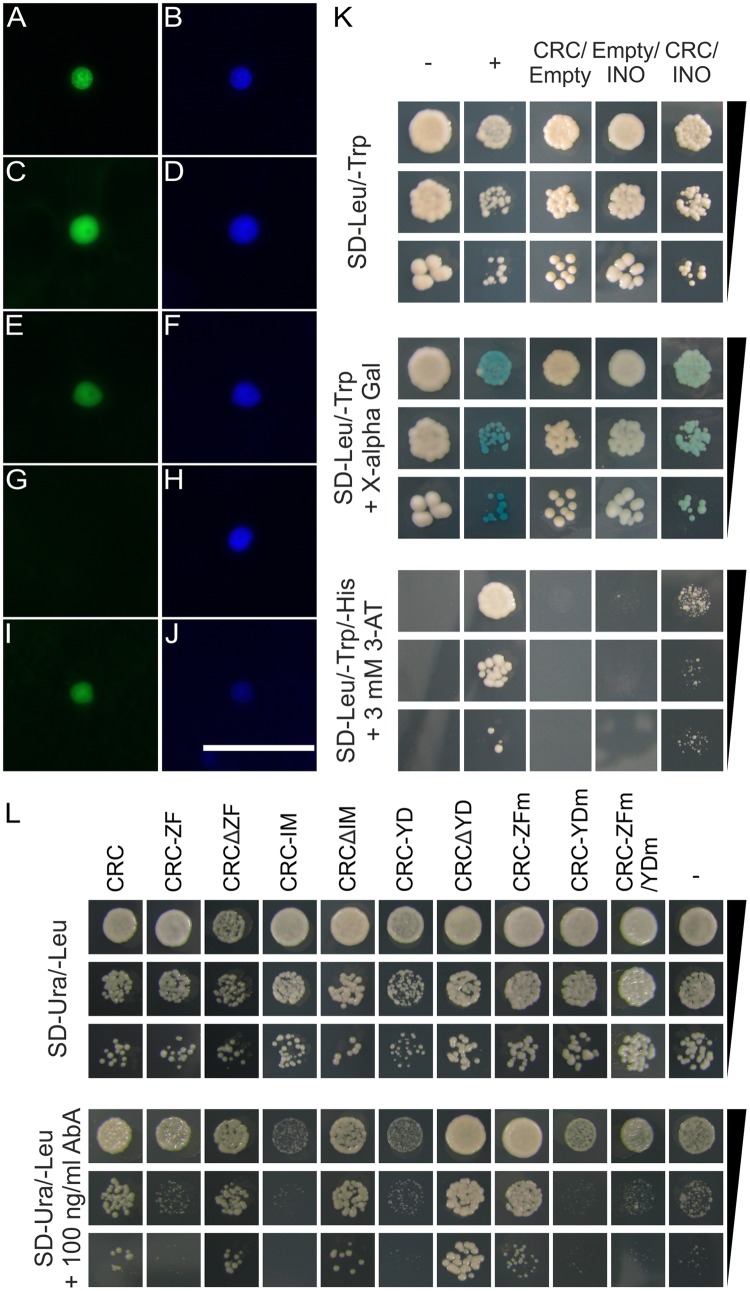
Protein interaction analysis of CRC and analysis of interacting domains. **(A–J)** Protein interaction analysis by BiFC using multiple CRC versions. YFP_C_ and YFP_N_ tagged full-length CRC, deletion version of CRC, and single domains were detected by fluorescence microscopy. **(A)** Combination CRC-YFP_C_-/CRC-YFP_N_ visualizing the YFP signal, **(B)** DAPI staining of the cell shown in **(A)**. **(C)** Combination YFP_C_-CRCΔZF/YFP_N_-CRCΔZF visualizing the YFP signal, **(D)**, DAPI staining of the cell shown in **(C)**. **(E)** Combination YFP_C_-CRCΔIM/YFP_N_-CRCΔIM visualizing the YFP signal. **(F)** DAPI staining of the cell shown in **(E)**. **(G)** Combination YFP_C_-CRCΔYD/YFP_N_-CRCΔYD visualizing the YFP signal. **(H)** DAPI staining of the cell shown in **(G)**. **(I)** Combination YFP_C_-CRC-YD/YFP_N_-CRC-YD visualizing the YFP signal. **(J)** DAPI staining of the cell shown in **(I)**. ZF, zinc finger domain; IM, intermediate domain; YD, YABBY domain. Scale bar represents 50 μm. **(K)** Y2H analysis of CRCs interaction with INO. Yeast cell suspensions of the respective test strains with an OD_600_ of 0.1, 0.01, and 0.001 plated on SD-Leu/-Trp medium and stained with X-α-Gluc after 5 days of incubation and on SD-Leu/-Trp/-His + 3mM 3-AT. As positive control, a combination of AD-EcSEI/BD-EcDEF2 (Lange et al., 2013) was used and a combination of the empty vectors pGADT7/pGBKT7 as negative control. **(L)** CRC’s DNA binding capabilities in an Y1H analysis. The *S. cerevisiae proKCS15* reporter strain was transformed with full length CRC, single domains, deletion constructs, and mutant versions, fused to the activation domain of GAL4. Yeast cell suspensions of the respective test strains with an OD_600_ of 0.1, 0.01, and 0.001 plated on SD-Ura/-Leu and on SD-Ura/-Leu + 100 ng/ml AbA. As negative controls, the *proKCS15* bait strain was transformed with an empty pGADT7 vector.

As other transcription factors with a HMG box or with only one zinc finger domain are known to form homo- and heterodimers (Yanagisawa, 1997; Sanchez-Giraldo et al., 2015), we hypothesize that CRC also forms dimeric complexes. We thus performed a BiFC with full-length CRC, CRC deletion constructs, and CRC single domains to elucidate the formation of homodimers and to identify the necessary domain for this protein–protein interaction. Additionally, an Y2H analysis was carried out in which we combined full-length AD-CRC/BD-CRC. A restoration of YFP fluorescence in the nuclei of *N. benthamiana* leaf cells by the formation of CRC homodimers was only observed when YFP_C_ and YFP_N_ tag were fused to CRC in the same orientation (**Figures 2A,B**). The same restriction in tag orientation was found in the other constructs such that homodimer formation was also observed when the deletion constructs CRCΔZF (**Figures 2C,D**) and CRCΔIM (**Figures 2E,F**) were combined, respectively. Here, the reconstituted YFP was localized to the nuclei, similar to the full-length CRC combinations, as the comparison with DAPI staining indicates. In contrast, combinations with the other single domains (Supplementary Figure 4) or with CRCΔYD (**Figures 2G,H**) failed to restore YFP fluorescence. Vice versa, only YABBY domain combinations were able to reconstitute the YFP signal when the individual domains were tested (**Figures 2I,J**). This indicates that the YABBY domain harbors the protein-protein interaction site. However, this YABBY domain mediated homodimerization did not occur in the Y2H analysis, where the combination of AD-CRC/BD-CRC was neither able to grow on histidine drop-out medium (SD- Leu/Trp/His), nor produced a blue staining after incubation with X-α-Gluc (Supplementary Figure 5 and Supplementary Table 2).

The majority of the YABBY proteins is able to form homo- and heterodimers with other YABBY proteins (Stahle et al., 2009) and we were interested to see if this is also the case for CRC. We performed an Y2H analysis of CRC and the remaining five *A. thaliana* YABBY proteins (FIL, YAB2, YAB3, INO, and YAB5) (**Figure 2K**, Supplementary Figure 5, and Supplementary Table 2), by combining AD-CRC with the respective BD-YABBY constructs and vice versa. Interestingly, only the combination AD-CRC/BD-INO showed an interaction indicated by weak growth on the SD-Leu/Trp/His + 3-AT plates and on the blue staining in the α-Gal assay (**Figure 2K**).

### The YABBY Domain Is Necessary for DNA Binding

In addition to the nuclear localization of a protein, the ability to bind DNA is a requirement for its action as a transcription factor. *In silico* analysis by Bowman and Smyth (1999), predicted that CRC exhibits two putative DNA binding domains: The N-terminal C2C2 zinc finger domain and the C-terminal YABBY domain, which has a helix-loop-helix structure with similarities to a HMG box. We used partial promoter regions of *KCS7* and *KCS15*, identified by Han et al. (2012), as direct targets of CRC, in an Y1H experiment to analyze which protein domain mediates the DNA binding. However, the *proKCS7* bait strain was discarded due to a high degree of autoactivation.

Full-length CRC, translationally fused to the activation domain of the yeast transcription factor GAL4, was able to bind to the promoter region of *KCS15* and enabled colony growth (colonies with > 1 mm) (**Figure 2L**) on SD-Ura/-Leu medium, supplemented with 100 ng/ml Aureobasidin A. When transforming the *proKCS15* bait strain with the respective single domains of CRC in this Y1H assay, well grown colonies (>1 mm) were observed only in the following combinations of the deletion constructs and *proKCS15* (**Figure 2L**): intermediate domain plus YABBY domain (CRCΔZF); zinc finger plus YABBY domain (CRCΔIM): and zinc finger plus intermediate (CRCΔYD). In contrast, no interaction could be shown when only the single domains (*proKCS15*/CRC-ZF, *proKCS15*/CRC-IM, and *proKCS15*/CRC-YD) are used in the analysis, suggesting that single domains alone are unable to mediate DNA binding. Interestingly, we did not observe differences in *KCS7* or *KCS15* gene expression in buds between wild type and *crc-1* plants (Supplementary Figure 8).

To further analyze this effect, we mutated CRC with site directed mutagenesis and rendered either the zinc finger (CRC-ZFm) or the YABBY domain (CRC-YDm) non-functional by changing two and nine amino acids, respectively. A third CRC version comprised a fusion of the two non-functional domains (CRC-ZFm/YDm). When these CRC versions were introduced into the *proKCS15* bait strain, only the CRC version with a mutagenized zinc finger (CRC-ZFm) grew well on the selection medium. In summary, we show that the intermediate domain does not participate in DNA binding. For sequence specific DNA binding, the YABBY and ZF domains are required, but if the ZF three-dimensional structure is abolished, DNA binding remains unaffected. However, if the YABBY domain’s 3D structure is destroyed, DNA binding is not possible anymore suggesting a more prominent role for the YABBY domain in CRC-DNA binding.

### CRC Acts as a Bifunctional Transcription Factor

**FIGURE 3:**
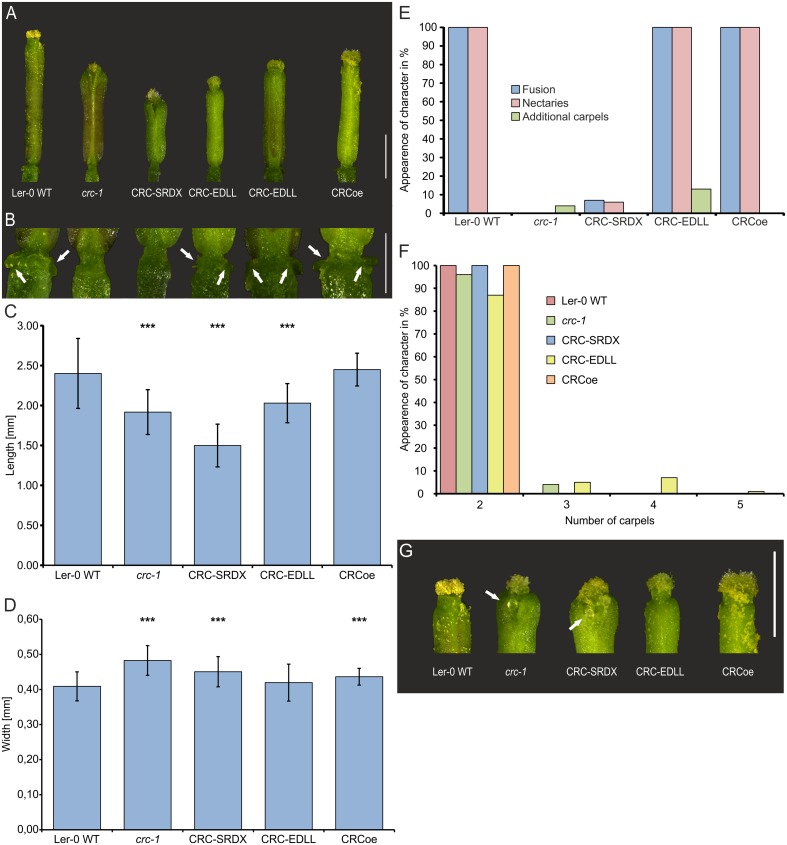
Phenotypic analysis of CRCoe, CRC-SRDX, and CRC-EDLL expressing *A. thaliana* Ler-0 plants. **(A)** Representative gynoecia of Ler-0 wild type, *crc-1*, proUBQ10:CRC (CRCoe), proUBQ10:CRC:SRDX, and proUBQ10:CRC:EDLL plants. Scale bar represents 1 mm. **(B)** Magnification of the gynophore region of the exemplary gynoecia with arrows highlighting the nectaries. Scale bar represents 500 μm. Statistical analysis of gynoecium length **(C)**, width **(D)**, a summary of other described defects of the *crc-1* phenotype **(E)**, and the number of carpels in the analyzed gyneocia of the four plant lines. In each line, except for CRCoe (*n* = 30), 100 randomly picked gynoecia were analyzed. Both, length and width comparisons **(C,D)** are mean values with their respective standard deviation. Percent values are shown in **(E,F)**. Student’s *t*-test was applied to compare the wild type gynoecia with the other lines and significant differences were marked with up to three asterisks (*p* < 0.001). **(G)** Magnification of the apical region of representative gynoecia of the respective lines showing protruding ovules (arrows). Scale bar represents 1 mm.

CRC’s role as a transcription factor relies not only on its ability to bind to DNA but also on the effect of this interaction. We were interested to answer the question if CRC acts as an activator or repressor of transcription or if CRC has a dual role in which it represses some developmental processes but activates others. Thus, CRC was transformed into a constitutive repressor of transcription by translational fusion of the SRDX domain to the CRC CDS. Consequently, molecular functions requiring transcriptional activation of target genes cannot be carried out by the fusion protein resulting in a mutant phenotype (**Figure 3**). Conversely, the translational fusion of the EDLL domain converted CRC in a constitutive activator of transcription, abolishing repressive function of CRC. The respective fusion constructs, driven by *A. thaliana UBQ10* promoter were transformed into *A. thaliana* Ler-0 and the plants were genotyped. For phenotypical analysis, 10 gynoecia of each of the 10 independent transgenic lines, except for CRCoe with only three transgenic lines and 30 analyzed flowers, were compared to equal numbers of Ler-0 and *crc-1* gynoecia (**Figure 3**). Wild type gynoecia had an average length of 2.40 ± 0.44 mm (**Figure 3C**), similar to CRCoe gynoecia (2.45 ± 0.21), whereas *crc-1* gynoecia were significantly shorter (1.92 ± 0.28 mm). Both, CRC-SRDX and CRC-EDLL, were significantly shorter than wild type gynoecia, with 0.45 ± 0.04 mm and 2.03 ± 0.24 mm respectively. Interestingly, the CRC-SRDX lines developed gynoecia even shorter than the *crc-1* plants. Gynoecia of *crc-1* (0.48 ± 0.04 mm), CRC-SRDX (0.45 ± 0.04 mm), and CRCoe (0.44 ± 0.02) plants were wider than gynoecia of wild type plants (0.41 ± 0.04 mm) and of CRC-EDLL plants with only two carpels (0.42 ± 0.05 mm) (**Figure 3D**). Additional to these growth parameters, the presence or absence of other known *crc-1* phenotypes (carpel fusion, nectary formation, and the presence of additional carpels) was analyzed in the three transgenic lines (**Figure 3E**). All analyzed gynoecia of wild type plants were completely fused in their apical region, exhibited nectaries at their base and had two carpels. In contrast, all gynoecia of *crc-1* plants were apically unfused, and failed to develop nectaries. Additionally, 4 % of the analyzed *crc-1* gynoecia consisted of more than two carpels. CRCoe gynoecia were identical with wild type gynoecia in terms of carpel number, apical fusion, and presence of nectaries. CRC-SRDX gynoecia failed in 93% to complete the apical fusion, their septa were partially unfused, and they showed a reduced amount of style and stigma tissue, similar to the *crc-1* phenotype. Moreover, ovules protruded from the inside of the carpels (**Figure 3G**) and nectaries were present in only 6% of the analyzed gynoecia but additional carpels were not observed.

CRC-EDLL gynoecia showed no defects in carpel or septum fusion and nectaries were present in all gynoecia. However, 13% of the analyzed gynoecia were composed of more than two, in most cases four carpels (**Figure 3F**). We then hypothesized that this phenotype may be dosage dependent and carried out qRT-PCR on plants over expressing CRC-EDLL with CRC-specific primers (Supplementary Figure 6). Interestingly, expression of CRC-EDLL does not significantly differ in the transgenic lines between plants developing a normal carpel number vs. plants with a surplus in carpels, suggesting that the EDLL domain has a stronger effect on downstream targets than the absence of CRC protein.

Taken together, these data suggest that CRC acts as a transcriptional activator in the regulation of carpel fusion and nectary development, in promoting longitudinal growth and restricting the width of the gynoecium. However, repression of target genes by CRC is required for floral meristem termination.

## Discussion

CRC is one of the major carpel developmental regulators in *Arabidopsis*. In this study, we have further characterized the protein by assigning functions to the different protein domains of CRC and showed that CRC acts as both, an activator and a repressor of transcription.

### The Cumulative Nature of the Nuclear Localization Signal

The localization studies of GFP::CRC and different CRC variants showed that CRC is a nuclear localized protein and is almost completely absent from the cytoplasm, which is similar to the subcellular localization of e.g., FIL and INO (Sawa et al., 1999; Meister et al., 2002). Required for this nuclear localization is the C-terminal YABBY domain (**Figures 1B–K**) but remarkably, the single amino acid mutagenesis of the predicted NLS, did not abolish the nuclear localization (Supplementary Figure 3). Successive deletion of the eight amino acids in the NLS only led to an accumulation of the GFP fusion protein in the cytoplasm suggesting an additive effect of these amino acids toward nuclear localization. Further, this finding may suggest the presence of NLS supporting amino acids in the YABBY domain allowing more efficient binding to certain IMPORTINα isoforms (Sankhala et al., 2017). However, some proteins such as the floral homeotic regulators AP3 and PI (McGonigle et al., 1996) or the mismatch repair system proteins MLH1 and PMS2 (Wu et al., 2003) must enter the nucleus as dimer, as only their dimerization leads to the reconstitution of a functional NLS. In our experiment, the construct with only the short NLS sequence (8 amino acids) is missing the largest part of the YABBY domain, and thus the GFP::CRC-NLS protein is most likely unable to homodimerize such that a functional NLS cannot be reconstituted.

At present, we cannot conclude from our data if the lack in dimerization, or missing NLS supportive amino acids are the reason for the failing nuclear import of CRC-NLS. Interestingly, an influx of the single domain GFP::CRC-ZF, GFP::CRC-IM, and the truncated GFP::CRCΔYD, GFP::CRC-YDΔNLS fusion proteins into the nuclei could be observed in combination with strong residual cytoplasmic localization (**Figure 1F** and Supplementary Figure 2). This is most likely due to the small size of the fusion proteins and the fact that the GFP fusion construct concentration is higher than the endogenous CRC concentration in wild type plants would be. Generally, nuclear pore complexes allow non-specific influx of proteins up to a size of ∼60 kDa (Haasen et al., 1999), hence, the fusion proteins are small enough (∼28–47 kDa, **Figure 1A** and Supplementary Figure 2A) to diffuse into nuclei. However, this diffusional influx yields to an active nuclear import if the entire YABBY domain is present in the respective fusion protein.

### The Conundrum of YABBY Protein DNA Binding

Two putative DNA binding domains, the N-terminal zinc finger and the C-terminal YABBY domain were suggested previously (Bowman and Smyth, 1999). Interestingly, our yeast-1-hybrid analysis using the *KCS15* promoter (Han et al., 2012) demonstrated that neither the zinc finger nor the YABBY domain alone binds sufficiently enough to cause transcription of the selective marker. Conversely, all deletion constructs mildly induced colony growth on selection medium, suggesting that one single domain alone is not able to bind to DNA, but cooperatively with other parts of CRC DNA binding is achieved (**Figure 2L**). The YABBY domain is most likely the main interacting domain, as we could show that mutations in the YABBY domain drastically reduce DNA binding (CRC-YDm and CRC-ZFm/YDm). These mutations disrupted the two long α-helices in the YABBY domain (Supplementary Figure 1), which are responsible for DNA bending and binding in HMG box proteins (reviewed in Malarkey and Churchill, 2012). Furthermore, the YABBY domain shows a high degree of conservation of those amino acid residues that are necessary for DNA binding in HMG boxes (Bowman and Smyth, 1999). In contrast, mutations in the zinc finger domain did not result in a loss of DNA binding of CRC, which is in line with observations from experiments using another YABBY protein (Smyth et al., 1990; Yang et al., 2016). These findings from YABBY proteins are similar to the DNA binding behavior of major mammalian cell cycle regulator and tumor suppressor protein p53 whose DNA binding capability of the central DNA binding domain additionally requires the C-terminal domain (Laptenko et al., 2015), hence, cooperative DNA binding seems a more generally applicable concept.

DNA binding activities of other YABBY proteins were analyzed previously with partially conflicting results: Whereas OsYABBY1 from *Oryza sativa*, specifically binds to a GA-responsive element in the promoter of *GIBBERELLIN 3-BETA-DIOXYGENASE 2* (*2GA3ox2*) (Dai et al., 2007), FIL has been described to bind unspecifically to DNA via its YABBY domain (Kanaya et al., 2002). However, a binding motif analysis via protein-binding microarrays (PBM) identified the DNA binding motifs of FIL and YABBY5 (Franco-Zorrilla et al., 2014). Interestingly, typical zinc finger DNA binding motifs were identified by chromatin immunoprecipitation DNA-sequencing (ChIP-Seq) of YABBY proteins from *Glycine max* (Shamimuzzaman and Vodkin, 2013). And, even more confusing, a recent study identified the YABBY domain as DNA interaction domain by binding to a typical zinc finger motif (Shamimuzzaman and Vodkin, 2013; Yang et al., 2016).

Hence, we propose a model in which CRC forms dimers mediated by the YABBY domain. This dimerization enables the YABBY domain and the zinc finger to individually bind to DNA, most likely to different DNA motifs as both, typical zinc finger DNA binding motifs and non-zinc finger DNA binding motifs have been identified for YABBY proteins (Shamimuzzaman and Vodkin, 2013; Franco-Zorrilla et al., 2014).

Our analyzes did not show a clear function for the intermediate domain, and phylogenetic analysis of different YABBY orthologs show a high variability of the domain (Yamada et al., 2011). Hence, we hypothesize that the intermediate domain, even though it is not necessary for homodimerization and the interaction with DNA, may act as a flexible linker (Supplementary Figure 1) between die DNA-binding YABBY domain and the zinc finger.

Our protein interaction analysis (**Figures 2A–K**) has shown that CRC forms homodimers and interacts with INO mediated by the YABBY domain. The YABBY domain is predicted to form two amphipathic helices (Supplementary Figure 1) with similarities to HMG boxes, the latter are also involved in dimerization between HMG box containing proteins (Roemer et al., 2008; Sanchez-Giraldo et al., 2015), suggesting conservation on sequence and two-dimensional structure level between classical HMB boxes and YABBY domains.

### CRC Acts as a Bifunctional Transcription Factor

Our analysis with the functionally modified CRC protein (**Figure 3**) shows that CRC exhibits characteristics of both, activator and repressor: It serves as an activator of target gene transcription required for carpel fusion, septum formation, and nectary development. Conversely, CRC represses transcription of target genes involved in the termination of the floral meristem. Furthermore, the transgenic lines showed enhanced phenotypes compared to the *crc-1* mutant, especially in terms of supernumerous carpels. The *crc-1* mutant exhibits up to three carpels at a low frequency. Carpel number can be increased by crossing *crc-1* with mutants of the redundant acting regulators of floral meristem termination (RBL, SQN, ULT1, and AG). We thus think that CRC-SRDX and CRC-EDLL may superimpose these regulatory pathways and increase the strength of their respective phenotypes [as shown before for other YABBY proteins by Bonaccorso et al. (2012), and as a general phenomenon by Tiwari et al. (2012), and Figueroa and Browse (2015)]. Interestingly, mild over expression of CRC via the *UBQ10* promoter resulted only in a minor increase in the width of the respective gynoecia compared to wild type gynoecia, but the gynoecia were still significantly thinner than gynoecia of *crc-1* or CRC-SRDX gynoecia. Hence, the overexpression of CRC via *proUBQ10* shows no adverse effects related to over expression like transgene silencing or co-suppression (Elmayan and Vaucheret, 1996; Elmayan et al., 1998; Mishiba et al., 2005) which might inhibit phenotypical analysis.

There is a growing number of examples for bifunctional transcription factors in animals (Willy, 2000; Bernadt et al., 2005) and in plants. E.g., the homeobox gene WUSCHEL activates early *AG* expression but otherwise acts as a repressor for *CLV3* expression to maintain stem cell identity and homeostasis in the shoot apical meristem and floral meristems (Lohmann et al., 2001; Leibfried et al., 2005; Ikeda et al., 2009) and APETALA 2 that activates floral repressors like AGAMOUS-LIKE 15 and represses floral activators such as SUPPRESSOR OF OVEREXPRESSION OF CONSTANS (Yant et al., 2010). So far, the *A. thaliana* members of the YABBY family, except for FIL and YAB3, show mainly repressive effects, as they form regulatory complexes with the corepressors LEUNIG and LEUNIG HOMOLOG and repress the transcription of e.g., EXPANSIN 11 (Navarro et al., 2004; Stahle et al., 2009; Bonaccorso et al., 2012). Also in rice, OsYAB1 and OsYAB4 are involved in the regulation of gibberellin levels by repressing *GA3ox2* expression (Dai et al., 2007; Yang et al., 2016). Additionally, Yamaguchi et al. (2017) found that CRC represses the expression of TORNADO 2, a plasma membrane localized protein involved in auxin homeostasis in the gynoecium during early flower development. Interestingly, FIL and YAB3 can also act as activators on the expression of AUXIN RESPONSE FACTOR 4 and KAN1. Furthermore, *BLADE ON PETIOLE 1* and *2* are, as *CRC*, necessary for nectary development and as CRC was reported to act epistatically on these two genes (McKim et al., 2008). Both genes may be direct target genes of CRC as their promoter regions exhibit multiple predicted YABBY binding sites in close proximity to their respective start codons (Supplementary Figure 7). Especially the putative YABBY binding motif (YBM) 4 (WATNATW, Franco-Zorrilla et al., 2014) appears frequently in *proBOP1* and *proBOP2* (28 times and 33 times, respectively). To rule out the possibility of random occurrence of this motif in any given stretch of DNA, we analyzed the promoter regions of the established qRT-PCR reference genes *POLYUBIQUITIN 10* (*UBQ10*), *GLYCERALDEHYDE – 3 - PHOSPHATE DEHYDRO-GENASE C – 2* (*GAPDH2*), and *ELONGATION FACTOR 1* α (*EF1*α) (Czechowski et al., 2005) and found YABBY binding motifs completely absent. However, further studies are required to experimentally corroborate these *in silico* predictions.

Siggers et al. (2014) showed that the conserved DNA binding motifs of a C2H2 zinc finger transcription factors can change in reaction to different protein interaction partners. Apparently, protein changes mediate binding to new target sites while not affecting the binding to a core set of common target sites. In the light of our observation combined with previously published results we hypothesize that CRC also binds to multiple DNA motifs via the zinc finger and/or the YABBY domain. However, all experiments carried out so far fail to show affinity to these binding sites in a quantitative way and it could well be that some experimentally identified binding sites require the interaction of CRC with specific co-factors not present in our yeast-based experiments. Because of the multitude of binding motifs, we hypothesize that CRC may use different DNA binding motifs in dependence of its interaction partners when activating vs. repressing transcription of target genes.

These two possibilities might explain the duality of YABBY proteins and especially of CRC, as certain functions might be dependent on the presence or absence of different protein interactors. Repression of transcription could be achieved through the interaction with the co-repressors LEU and LUG which are part of the regulatory network of abaxial-adaxial determination of lateral organs (Stahle et al., 2009). Additional interaction partners were identified in a high-throughput protein-protein interaction screen of CRC by Trigg et al. (2017): NGATHA4 or TEOSINTE BRANCHED1-CYCLOIDEA-PCF15 (TCP15), transcription factors involved in style development via regulation of the of auxin biosynthesis (Trigueros et al., 2009; Lucero et al., 2015).

Activation of transcription may be mediated by the interaction of CRC with INDETERMINATE DOMAIN15, which is involved in auxin biosynthesis and auxin homeostasis during lateral organ development (Cui et al., 2013; Trigg et al., 2017). It forms complexes with the co-activators ANGUSTIFOLIA3 and GROWTH-REGULATING FACTOR2, which are both involved in carpel development (Lee et al., 2018; Trigg et al., 2017) and auxin homeostasis (Vercruyssen et al., 2014; Schuster et al., 2015). A tight link between CRC function and auxin has been established previously as the apical defects in the *crc-1* gynoecium can be rescued by exogenous auxin application (Ståldal et al., 2008).

Taken together, CRC is a bifunctional transcription factor that regulates carpel fusion and nectary formation by activating the expression of target genes and terminates the meristematic activity of the floral meristem by repression of target gene expression. To fulfill its functions, it forms homo- and heterodimers via the YABBY domain which is also the main DNA interacting domain and harbors also the NLS. CRC is directly regulated by multiple MADS box proteins (Lee et al., 2005; Ó’Maoiléidigh et al., 2013) and is tightly integrated in the carpel developmental network by interactions with many auxin regulating proteins (Ståldal et al., 2008; Yamaguchi et al., 2017). Previously, it was shown that the function of *CRC’s* orthologs from only distantly related species can be very diverse, such that the rice ortholog *DL* is also involved in leaf midrib formation and carpel organ identity (Yamaguchi et al., 2004). *EcCRC* from *E. californica* is essential for placental development, ovule initiation and lateral carpel margin formation (for a more comprehensive overview of CRC ortholog functions, see Orashakova et al., 2009). It is astonishing how a comparatively small and highly conserved transcription factor can take up many different functions in diverse plant lineages and one may wonder how this is achieved. We and others have shown that the *A. thaliana* CRC protein has multiple target sequences and our work indicates that it represses transcription of some while activates transcription of other target genes, most likely depending on its protein interaction partners. CRC orthologs from other angiosperm species may also have these flexible properties and may interact with a different set of proteins. This may, at least in part, explain the functional versatility of these transcription factors in the angiosperms analyzed to date.

## Author Contributions

TG carried out the all the experiments, except for the Y1H analysis, in which SB was heavily involved. TG and AB analyzed the data. TG and AB drafted the manuscript. AB developed the project.

## Conflict of Interest Statement

The authors declare that the research was conducted in the absence of any commercial or financial relationships that could be construed as a potential conflict of interest.
