# Supplementary material for: CRABS CLAW Acts as a Bifunctional Transcription Factor in Flower Development
Source: Front Plant Sci. 2018 Jun 20;9:835. doi: 10.3389/fpls.2018.00835 (PMC6019494; doi:10.3389/fpls.2018.00835)
Supplement: Supplementary file 1 [file Table_1.DOCX]

Supplemental Table 1: Sequences of the primers used in this study

| Primer | Sequence (5´-3´) |
| --- | --- |
| AtCRC Fw EcoRI | AAA AGA ATT CAT GAA CCT AGA AGA GAA ACC AAC C |
| AtCRC Rv BamHI | AAA AGG ATC CTC ACT TCT TCT CAC CGA A |
| AtCRC Fw BamHI | AAA AGG ATC CAT GAA CCT AGA AGA GAA ACC AAC C |
| Adapter ZF-YD Fw | CGG TGA AAT GCG GCC ATT GTG TCA AAC CTC CTG AGA AGA AG |
| Adapter ZF-YD Rv | CTT CTT CTC AGG AGG TTT GAC ACA ATG GCC GCA TTT CAC CG |
| Adapter ΔNLS Fw | CAC CTC CCT TTG TCG TCC TCC CAT CTG CAT ACA ACC |
| Adapter ΔNLS Rv | GAC GAC AAA GGG AGG TGG GTT GTA TGC AGA TGG GAG |
| Zincfinger Fw EcoRI | ACA TGA ATT CTG TAG CAT CTG CAA CAC CAT |
| Zincfinger Rv BamHI | TTT GGA TCC ACA ATG GCC GCA TTT CAC |
| Zincfinger Fw XbaI | ACA TTC TAG ATG TAG CAT CTG CAA CAC CAT |
| Intermediate Fw EcoRI | ACA TGA ATT CGG TAA CCT CTC GTT TCT C |
| Intermediate Rv BamHI | AAA GGA TCC CGA CAA AGG GAG GTG AG |
| Intermediate Fw XbaI | ACA TTC TAG AGG TAA CCT CTC GTT TCT C |
| Yabby Fw EcoRI | ACA TGA ATT CGT CAA ACC TCC TGA GAA G |
| Yabby Rv BamHI | TAT GGA TCC CTT AGC CCA ATT TTT GGC A |
| Yabby Fw XbaI | ACA TTC TAG AGT CAA ACC TCC TGA GAA G |
| C-CRC Fw BsaI | AACAGGTCTCAGGCTCAATGAACCTAGAAGAGAAACCAACC |
| C-CRC Rv BsaI | AACAGGTCTCACTGAACACTTCTTCTCACCGAATCC |
| pKCS15 Fw HindIII | ACA TAA GCT TCC AAT CAG GGA TGA AAC ATC |
| pKCS15 Rv SacI | ACA TGA GCT CAT AAT CAA ATA AAT ATG AGT CAG TG |
| pKCS7 Fw HindIII | ACA TAA GCT TCT CTC AAG CTA GCA TTA TGC |
| pKCS7 Rv SacI | ACA TGA GCT CGA TCA AGA GTT TTG TTT TTG TGG C |
| D-SRDX Top | AACAGGTCTCATCAGCACTGGATCTGGATCTGGAACTGCGTCTGGGCTTTGCGTGACTGCTGAGACCTGTT |
| D-SRDX Bottom | AACAGGTCTCAGCAGTCACGCAAAGCCCAGACGCAGTTCCAGATCCAGATCCAGTGCTGATGAGACCTGTT |
| D-EDLL Top | TCAGAAGAAGTTTTCGAGTTTGAGTATTTGGACGATAAGGTTCTTGAAGAACTTCTTGATTCAGAAGAAAGGAAGAGATAA |
| D-EDLL Bottom | GCAGTTATCTCTTCCTTTCTTCTGAATCAAGAAGTTCTTCAAGAACCTTATCGTCCAAATACTCAAACTCGAAAACTTCTT |
| NLS K110T Fw | CCTCCCTTTGTCGTCACACCTCCTGAGAAGAAG |
| NLS K110T Rv | TGAGGGAGATGGGGGCTG |
| NLS P111A FW | TTGTCGTCAAAGCTCCTGAGAAGAAGCAG |
| NLS P111A Rv | AGGGAGGTGAGGGAGATGg |
| NLS P112A Fw | TGTCGTCAAACCTGCTGAGAAGAAGCAGAGG |
| NLS P112A Rv | AAGGGAGGTGAGGGAGATG |
| NLS E113G Fw | GTCGTCAAACCTCCTGGGAAGAAGCAGAGGCTC |
| NLS E113G Rv | AAAGGGAGGTGAGGGAGATG |
| NLS K114T Fw | GTCAAACCTCCTGAGACGAAGCAGAGGCTCCCA |
| NLS K114T Rv | GACAAAGGGAGGTGAGGGA |
| NLS K115T FW | AAACCTCCTGAGAAGACGCAGAGGCTCCCATCT |
| NLS K115T Rv | GACGACAAAGGGAGGTGAG |
| NLS Q116L Fw | CCTGAGAAGAAGCTGAGGCTCCCATCTGC |
| NLS Q116L Rv | AGGTTTGACGACAAAGGGAG |
| NLS R117T Fw | CCTGAGAAGAAGCAGACGCTCCCATCTGCATAC |
| NLS R117T Rv | AGGTTTGACGACAAAGGGAGG |
| pKCS15 YB4mut rv | GCAATGCAAATAAATATGAGTCAGTGTAAC |
| pKCS15 YB4mut fw | AGCTCGGTACCCGGGGATC |
| pKCS15 YB3mut fw | TTCAACGGTAACATGCAGCAAAG |
| pKCS15 YB3mut rv | TGCTATGGTTTTGCTGCATCATTCAGTTGG |
| YB4-2 mut Rv | CAGCAAAACCATAGCATTCAACGG |
| YB4-2 mut Fw | CGACTATCAGTTGGTGTGGTGTAACTAAACC |
| CRC YDmut 1 Rv | GAAGCGGGGGTATGGAGATGGGAGCCTCTGCTTCTTC |
| CRC YDmut 1 Fw | ATGAGGGATGAGATCCAACGC |
| CRC YDmut 2 Rv | GATGGGTTGGATCGGATCCCTCGGGAAGCGGGGGTATGG |
| CRC YDmut 2 Fw | AAAAGTGCCAATCCGGAAATACC |
| CRC YDmut 3 Rv | GGCAGGAGCACTGGGAGCTTCACGGTGTGG |
| CRC YDmut3 Fw | AAAAATTGGGCTAAGTACATACCC |
| CRC YDmut4 Rv | GTACGGAGCCCAAGGTTTGGCAGGAGCACT |
| CRC YDmut4 Fw | ATACCCAACTCTCCTACTTCC |
| CRC ZFmut Rv | CCAGATGCTCCACCGGACGTAGTAGAGATGTTCGG |
| CRC ZFmut Fw | AACACCATCCTCGCGGTTGG |
| Adapter CRC ZF - pUC | TTGTAAAACGACGGCCAGTGGAATTCATGAACCTAGAAGAGAAACCAACC |
| Adapter CRC ZF - CRC YD | GAGGTTTGACACAATGGCCGCATTTCAC |
| Adapter CRC YD - CRC ZF | CGGCCATTGTGTCAAACCTCCTGAGAAGAAG |
| Adapter CRC YD - pUC | CTATGACCATGATTACGCCAGGATCCTCACTTCTTCTCACCGAATC |
| Adapter CRC ZF/IM - YDd | CAGATGGGAGGACGACAAAGGGAGGTGAG |
| Adapter CRC YDΔNLS - ZF | CTTTGTCGTCCTCCCATCTGCATACAAC |
| AtCRC_qPCR_fw | CTCTCGTTTCTCACCACAACTC |
| AtCRC_qPCR_rv | GCTTCTTCTCAGGAGGTTTGAC |
| RTq-At-Actin_Fw | AGTGGTCGTACAACCGGTATTGT |
| RTq-At-Actin_Re | GATGGCATGGAGGAAGAGAGAAAC |
| qPCR KCS15 Fw | AACTTATGCAGCTAGTAAGAACCC |
| qPCR KCS15 Rv | GTTCAAAGGAAGGCTCAAGAC |
| qPCR KCS7 Fw | ATTCTGTCCTTCTCCATCCA |
| qPCR KCS7 Rv | TAACAGCTTCCATACTCAACAC |
