# Supplementary material for: CRABS CLAW Acts as a Bifunctional Transcription Factor in Flower Development
Source: Front Plant Sci. 2018 Jun 20;9:835. doi: 10.3389/fpls.2018.00835 (PMC6019494; doi:10.3389/fpls.2018.00835)
Supplement: Supplementary file 2 [file Table_2.DOCX]

Supplemental Table 2: Summary of Y2H results. All combinations used in this study (to test for homo- and heterodimerization) are shown. +: yeast did grow on medium/showed α-galactosidase activity; +/-: faint growth on medium/faint α-galactosidase activity; -: did not grow on medium/no α-galactosidase activity.

| Combination | Growth on SD-Leu/-Trp | α-X-Gal staining | Growth on SD-Leu/-Trp/-His + 3 mM 3-AT |
| --- | --- | --- | --- |
| E/E (neg. control) | + | - | - |
| EcSEI/EcDEF2 (pos. control) | + | + | + |
| E/FIL | + | - | + |
| E/YAB2 | + | - | - |
| E/YAB3 | + | - | - |
| E/INO | + | - | - |
| E/YAB5 | + | - | - |
| CRC/E | + | - | - |
| CRC/FIL | + | +/- | + |
| CRC/YAB2 | + | - | - |
| CRC/YAB3 | + | +/- | - |
| CRC/INO | + | + | + |
| CRC/YAB5 | + | - | - |
| CRC/CRC | + | +/- | - |
