# Supplementary figures and images for: CRABS CLAW Acts as a Bifunctional Transcription Factor in Flower Development
Source: Front Plant Sci. 2018 Jun 20;9:835. doi: 10.3389/fpls.2018.00835 (PMC6019494; doi:10.3389/fpls.2018.00835)

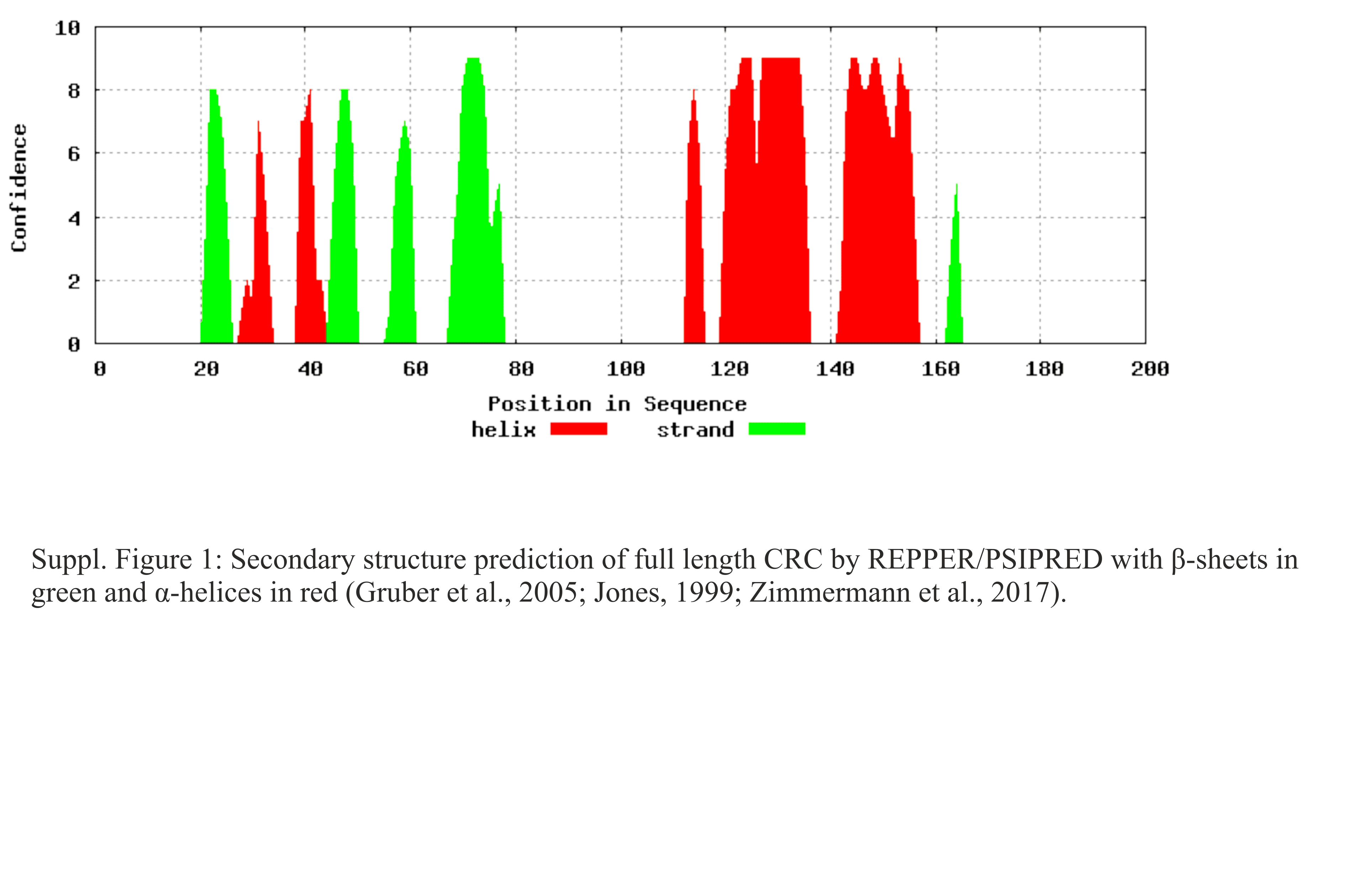

Supplement: Supplementary file 3 [file Image_1.TIF]

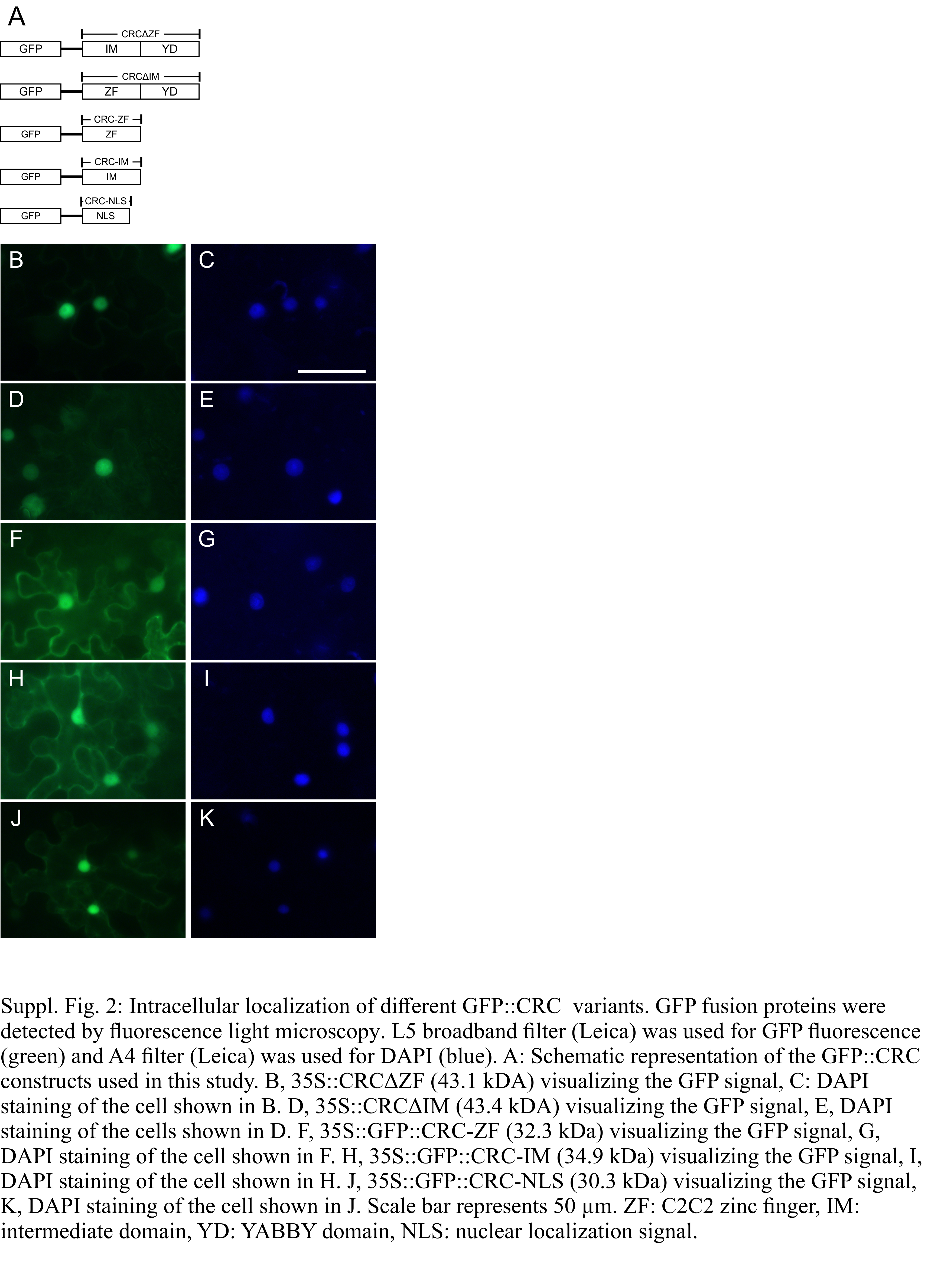

Supplement: Supplementary file 4 [file Image_2.TIF]

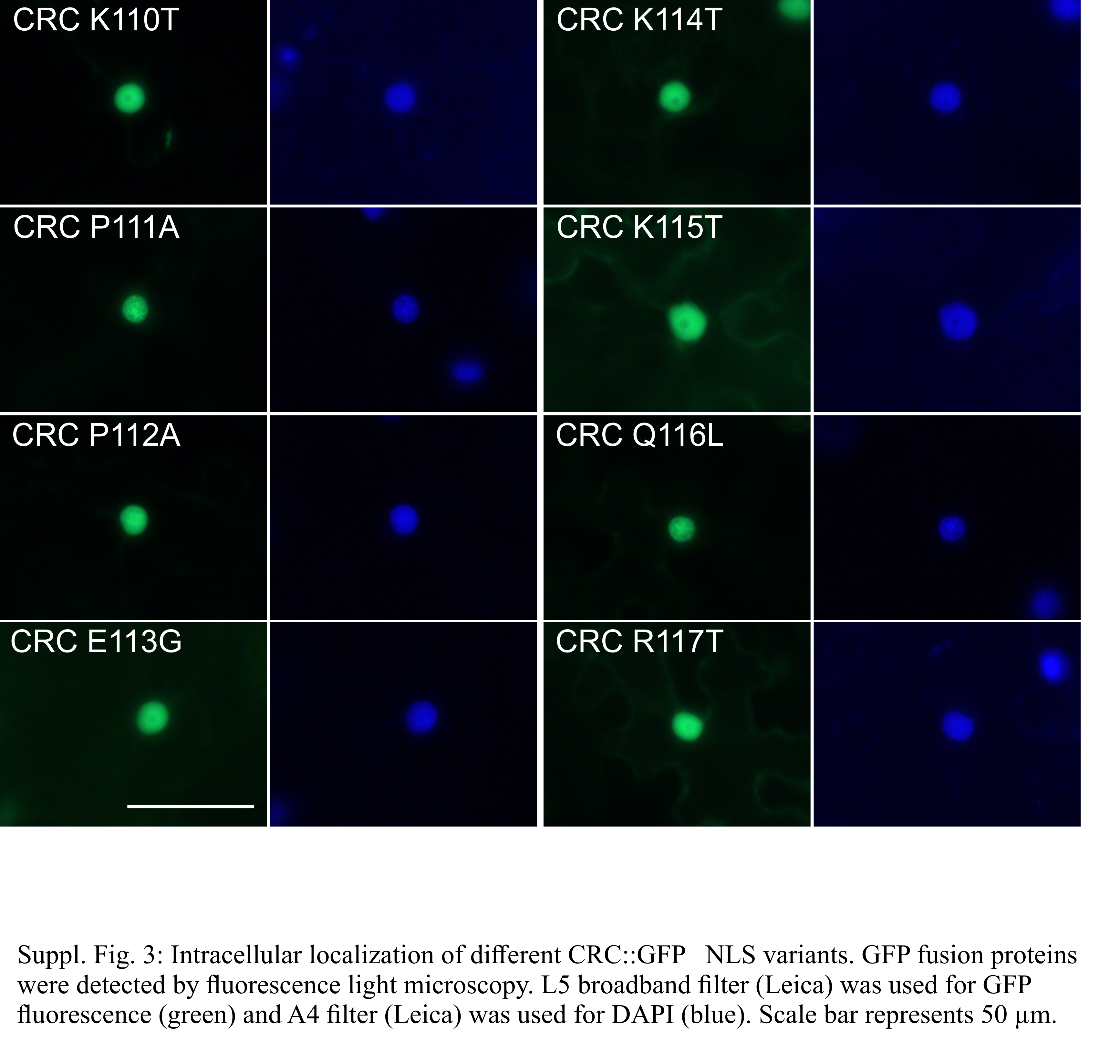

Supplement: Supplementary file 5 [file Image_3.TIF]

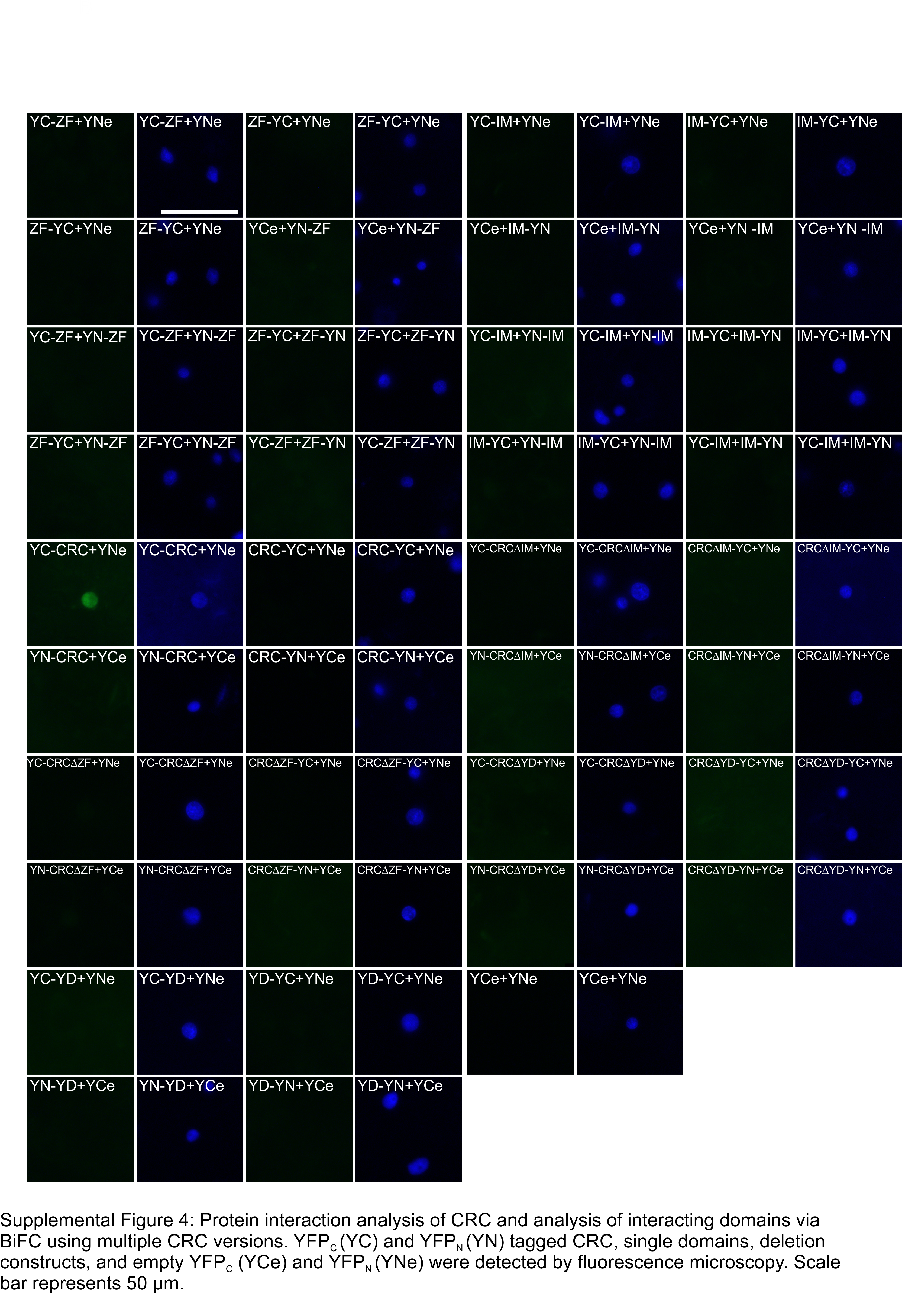

Supplement: Supplementary file 6 [file Image_4.TIF]

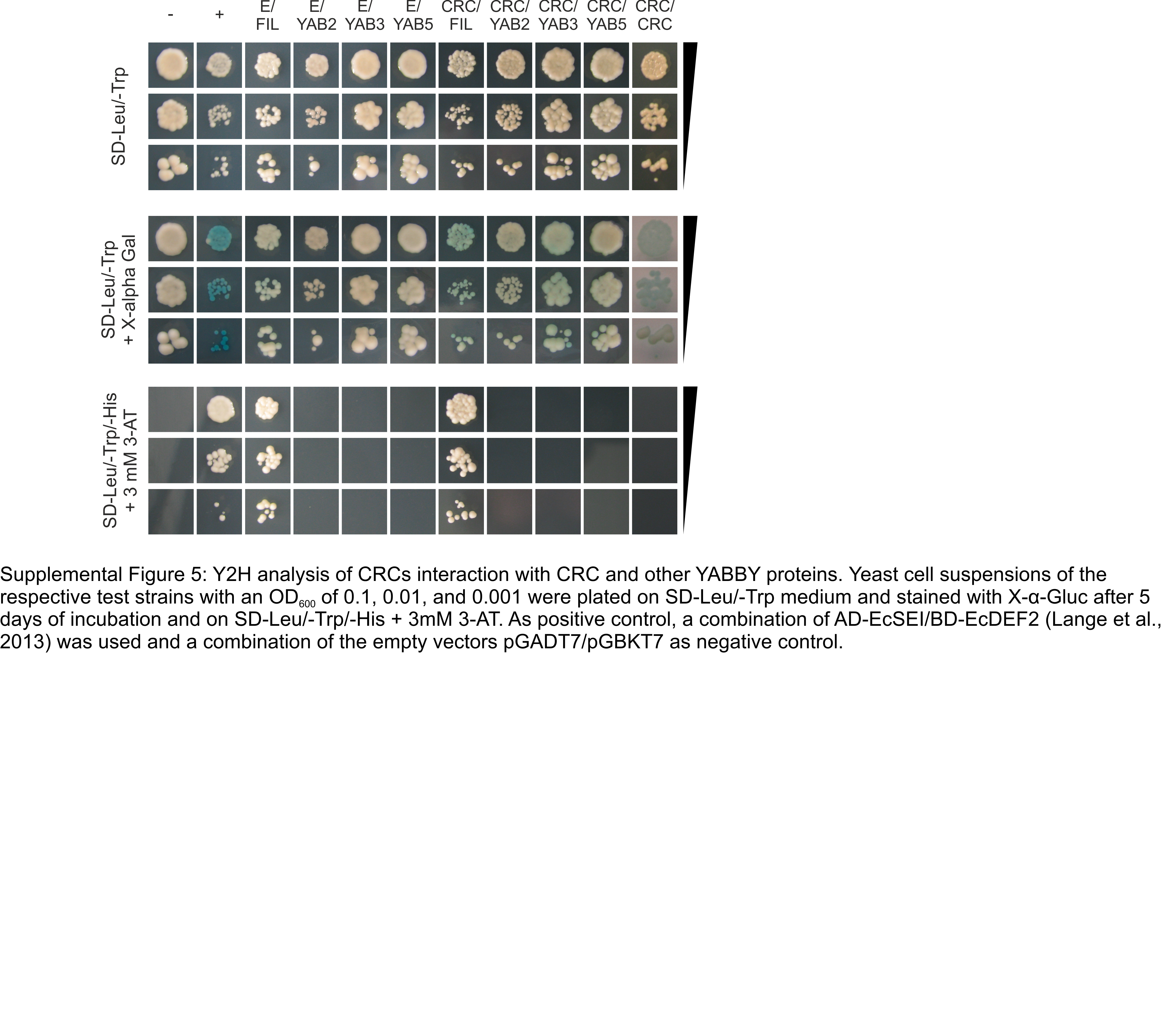

Supplement: Supplementary file 7 [file Image_5.TIF]

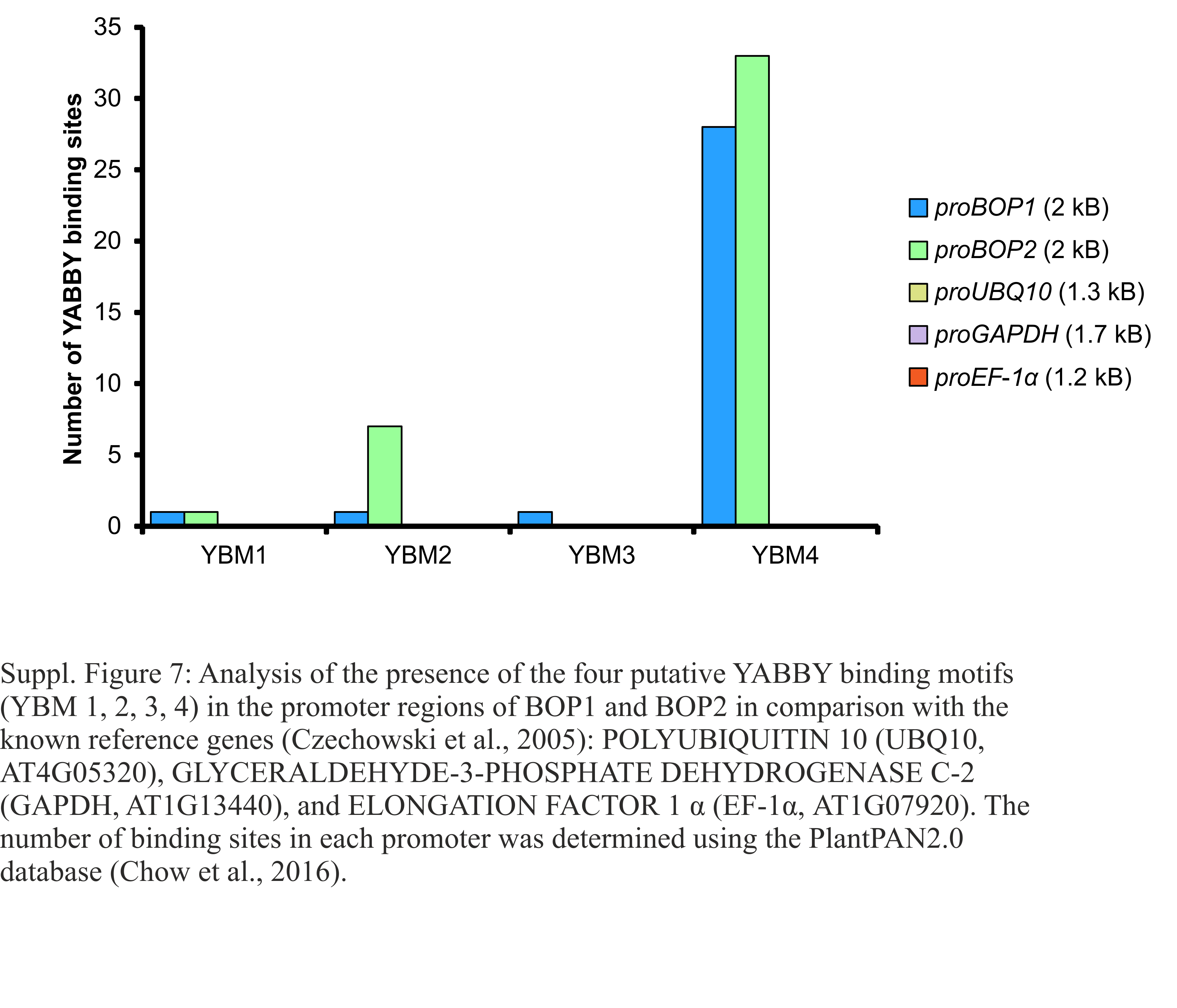

Supplement: Supplementary file 9 [file Image_7.TIF]

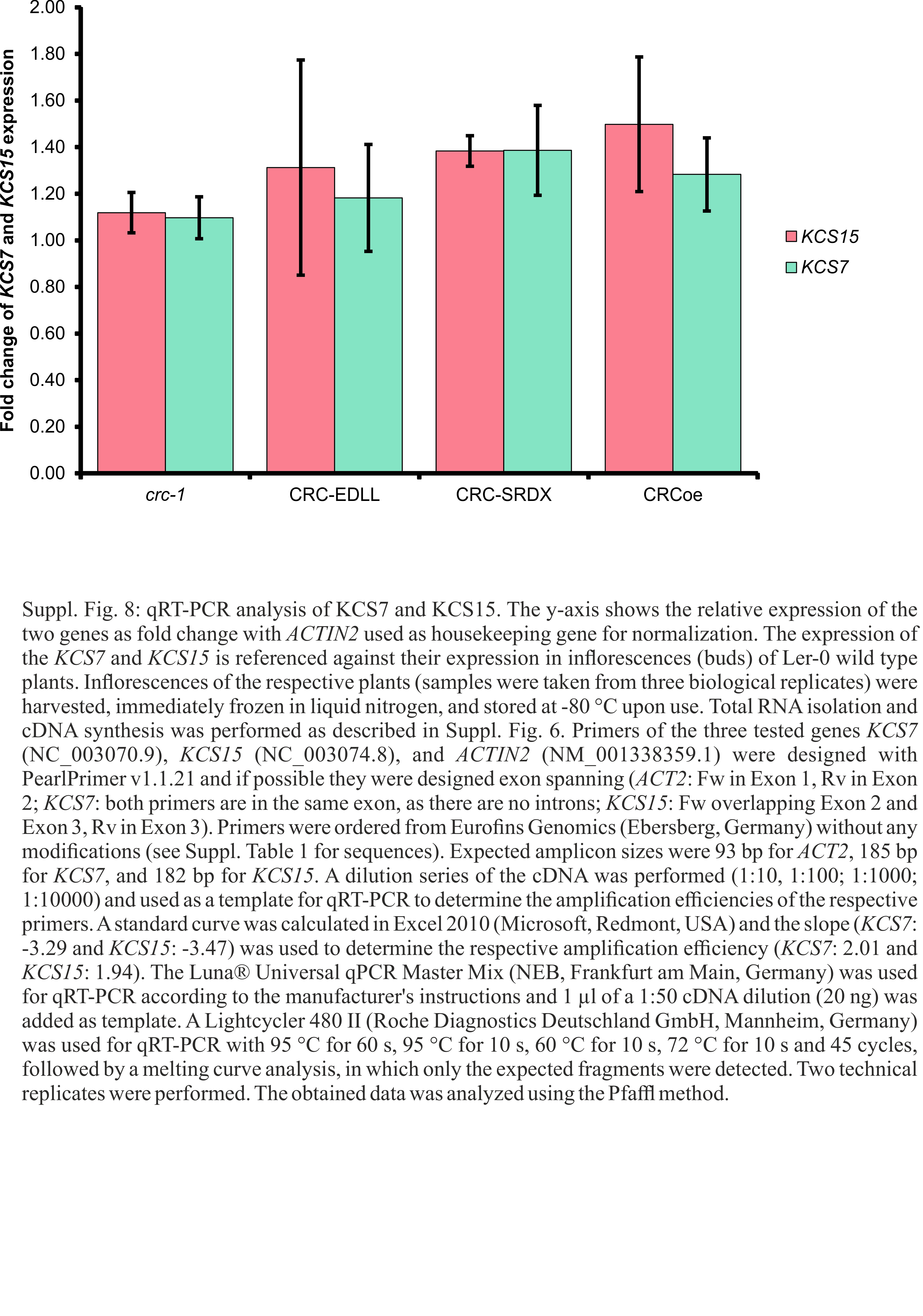

Supplement: Supplementary file 10 [file Image_8.TIF]
